# Acute Pulmonary Schistosomiasis: Computed Tomography (CT) Findings

**DOI:** 10.4269/ajtmh.2010.09-0425

**Published:** 2010-03

**Authors:** Gabrielle Weber-Donat, Nicolas Donat, Jacques Margery

**Affiliations:** Service de Radiologie, Service d'Anesthésie-Réanimation, Service de Pneumologie, Hôpital d'Instruction des Armées Percy Clamart Cedex, France

**Figure 1.**
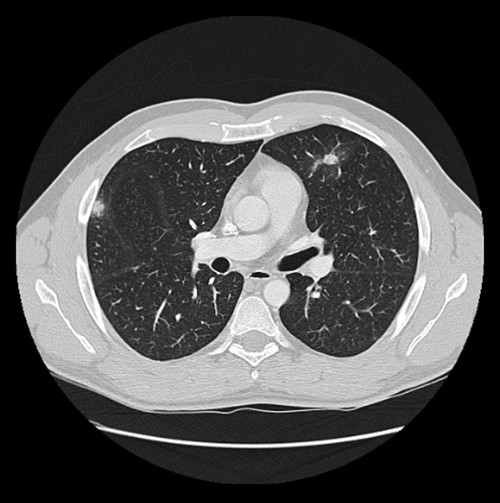
Computed tomography: macro nodules with ground-glass halo.

**Figure 2.**
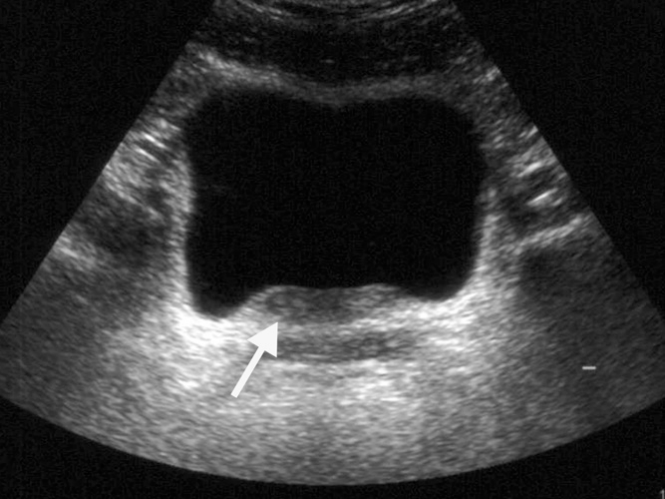
Ultrasonography shows thickening of the wall of the urinary bladder.

A 32 year-old white man, on his return from working in Nigeria, came for an examination of 3 weeks dry cough associated with fever and asthenia. He used to swim in fresh water in the Niger River valley for 6 months. There was no evidence of malaria. Hypereosinophilia at 18.8% was found in the blood (white blood cell [WBC] = 8,399/mm^3^, eosinophils cells = 1,579/mm^3^) and in broncho-alveolar lavage fluid (15%). Chest computed tomography (CT) showed bilateral micro- and macronodules with ground-glass halos (Figure 1). Strongly positive enzyme-linked immunosorbent assay (ELISA) confirmed schistosiomasis diagnosis without histological confirmation. Urine analysis discovered an unknown microscopic hematuria and ultrasonography revealed thickening of the bladder's wall (Figure 2). These anomalies suggest the diagnostic of schistosomiasis hematobium.

Schistosomiasis is a helminthic infection endemic to tropical and subtropical regions, acquired through direct contact with contaminated fresh water. Early schistosomiasis can occur 14 days after primary infection or heavy re-infection and reflects immunogenic response to migrating schistosomae cercariae before egg laying.^1^ This pulmonary phase is common even in the absence of clinical symptoms, regardless of the schistosomasis species (most often in *S. mansoni* and *S. japonicum*) and concerned almost always with non-immune travelers in schistosomiasis hematobium and mansoni.^1^ Computed tomography is the most sensitive exploration and shows typically pulmonary micro- or macronodules surround by ground glass halo. This ground-glass halo may be correlated with eosinophilic lung parenchyma infiltration or immune complex deposition.^2^
